# An Observational Retrospective Study Provide Information on Hospitalization and Severe Outcomes of the 2009 A(H1N1) Infection in Italy

**DOI:** 10.4021/jocmr883w

**Published:** 2013-06-21

**Authors:** Anna Maria Iorio, Barbara Camilloni, Michela Basileo, Lorenzo Monaldi, Enrica Lepri, Mariella Neri, Maura Marcucci, Franco Baldelli

**Affiliations:** a Dept. of Medical and Surgical Spec. and Public Health, University of Perugia, Via del Giochetto, 06122 Perugia, Italy; b Dept. of Exper. Med. and Bioch. Sc., University of Perugia, Via G. Dottori, 06121 Perugia, Italy; c Dept. of Internal Medicine, University of Perugia, Via G. Dottori, 06121 Perugia, Italy

**Keywords:** (H1N1) influenza virus, Pandemic, Hospitalization, ICU admission, Risk factors

## Abstract

**Background:**

The aim of this study was to try to ascertain whether, in the absence of a pre-organized programme, locally collected data might provide information about the epidemiological and clinical characteristics of the recent A(H1N1) pandemic in Italy.

**Methods:**

The study was an observational retrospective analysis of the clinic-epidemiological features performed by reviewing medical charts from 141 hospitalized patients with laboratory confirmed pandemic A(H1N1) infection in Umbria, a region of central Italy, in the period July 2009 to March 2010.

**Results:**

The pandemic virus was found capable of inducing severe illness requiring hospitalization or intensive care unit admission (ICU), or resulting in death. Age and comorbidity were found to be potential risk factors for severe disease. The mean age of the hospitalized patients was 37 years (range 0 - 93 yrs), however the mean age of ICU admitted patients, including people who did not survive, was higher as compared with those admitted to general medical ward (54 vs 35 yrs). The highest incidence of hospitalization was observed in the youngest group (0 - 17 yrs), the greatest rate of ICU admission in adults (18 - 64 years), and the risk of death in the oldest population (≥ 65 yrs). Comorbity conditions were present in some (55%), but not all hospitalized patients and increased with the age and the severity of the illness.

**Conclusions:**

The data obtained are compatible with the identified epidemiological characteristics of the A(H1N1) pandemic derived from partial information previously collected in Italy and from studies conducted in other European and non European countries. The results of our retrospective observational study suggest that locally organized data collection may give information on the epidemiological and clinical characteristics of a pandemic that are compatible with those obtained from more complex and complete studies.

## Introduction

New influenza viruses with pandemic or pandemic threat characteristics has frequently been emerged in the recent years. Since 1997, avian H5N1 influenza A viruses have sporadically infected humans, causing serious disease and high lethality [1]. In 2009, the emergence and the rapid global spread of the new swine-origin influenza A(H1N1) virus led the World Health Organization to declare a pandemic [2]. More recently, in October 2011, a limited human-to-human transmission of swine-origin triple reassortant influenza A(H3N2) has been reported [3].

Since the severity of a pandemic may vary from pandemic to pandemic, country to country and among different population groups, and because of the difficulties of foreseeing the emergence of new pandemic viruses, it is important to evaluate if results obtained on examining a restricted number of infected people retrospectively can be used to evaluate the general characteristics of a pandemic in a country. The epidemiological and clinical characteristics of the recent A(H1N1) 2009 pandemic, information on the clinical spectrum of illness and risk factors for severity among people who were hospitalized due to pandemic A(H1N1) influenza were collected in many countries on the basis of differently organized reporting systems [4, 5].

In Italy, the only published data available are those relative to the population showing influenza like illness in the pandemic months [6], to hospitalized paediatric population living in North Italy [7, 8] and to patients admitted to some Italian intensive care units (ICU) with extracorporeal membrane oxygenation capability [9]. In the absence of surveillance data collected for laboratory confirmed hospitalized cases during the whole pandemic period for the Italian population, the aim of this study was to try to ascertain whether locally organized data collection might provide information for Italy that is compatible with the identified epidemiological characteristics derived from the partial information previoursly collected and from more complex and complete studies in other countries.

The study was an observational retrospective analysis of the clinic-epidemiological features of hospitalized patients with laboratory confirmed pandemic A(H1N1) influenza virus infection, in Umbria, a region of central Italy, occurring between July and December 2009. The data were obtained by reviewing medical charts from 141 hospitalized patients with laboratory confirmed A(H1N1) infection, by distinguishing two levels of disease severity according to the type of ward they were referred to, namely, general medical ward (GMW) or ICU. In particular, we examined differences in patient characteristics and outcomes according to age classes.

## Methods

### Notification system and laboratory confirmation

In Italy, since July 2009, all hospitalized patients with suspected influenza infection were reported and tested for the presence of the new A(H1N1) virus utilizing the nationwide virological influenza surveillance network, INFLUNET (WHO National Influenza Centre (NIC) and regional collaborating laboratories) [6]. In Umbria, nasal and/or oropharyngeal samples from hospitalized patients with symptoms compatible with influenza virus infection (fever > 38°C and at least one respiratory and one generic symptom) were examined for the presence of the new A(H1N1) virus using the CDC validated real-time RT-PCR protocol [10] in the regional INFLUNET laboratory of Umbria, Italy. A confirmed case patient was defined as a person with symptoms compatible with influenza virus infection requiring hospitalization, and with laboratory confirmed 2009 pandemic A(H1N1) virus infection.

### Data collection

For each confirmed case patient admitted to hospital between July 2009 and March 2010, when patient’s clinical record could be accessed (based on the Legislative Decrete n. 196/2003, the need for a priori informed consent being waived because of the non-interventional study design), a medical chart review was retrospectively conducted using a standardized data collection instrument including demographic characteristics. Medical conditions that conferred a higher risk of influenza complications, clinical signs and symptoms, select laboratory tests, radiographic findings, and treatment course were all examined. All diagnostic testing was clinically driven.

### Statistical analysis

All data, extracted from medical charts, were allocated in an ad hoc database (Microsoft Excel, New Mexico, US) and used in the subsequent steps of the analysis. Simple descriptive epidemiology measures (mean and frequency expressed as a percentage) were used to describe the population. Univariate analysis was used to compare demographic details, comorbidity, clinical signs and symptoms, laboratory and radiological findings, clinical course and treatment of the patients admitted to either GMW or ICU. A multivariate logistic regression model was used to identify factors associated to the admission to ICU and also to explore the relationship between clinical complications and death.

## Results

**Figure 1:**
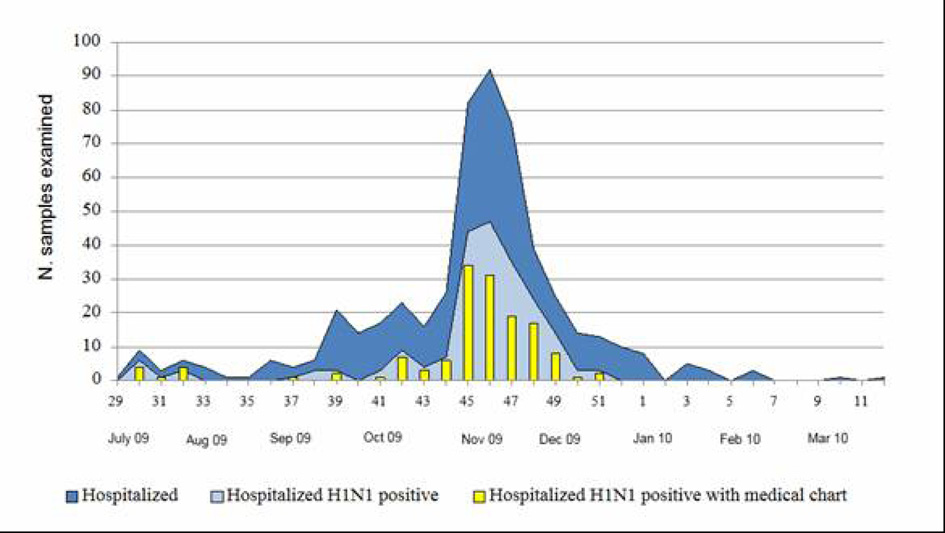
Week distribution of hospitalized patients with suspected influenza infection and of patients with laboratory confirmed 2009 pandemic A(H1N1) virus infection (July 2009 - March 2010).

During the period July 2009 - March 2010, 530 patients were recovered for suspected influenza in four different hospitals of Umbria, a region of central Italy with a population of about one million of people. The number of hospitalization peaked during weeks 45 - 49 in 2009 (Fig. 1). All the hospitalized people were laboratory tested for 2009 pandemic A(H1N1) influenza virus infection and the first laboratory confirmed case was identified on July 21st and the last on December 16th. Two hundred and ten of the 530 hospitalized patients (40%) were found to be positive for the presence of the 2009 pandemic influenza A(H1N1) virus. For 141 (67%) of these 210 patients we obtained data from the medical charts by a standardized data collection instrument. Of the 141 patients studied, 14 (10%) were admitted to ICU and 6 of them (43%) died. Considering the incidence per 100,000 inhabitants, using the estimated population of Umbria for 2009 and subdividing the 141 patients in three age groups, the data reported in Table 1 show that the overall hospitalization rate was 14.8 per 100,000 inhabitants (22.1, considering the overall 210 patients with a positive A(H1N1) laboratory test) with the highest rate in the children aged 0 - 17 (34.4). The incidence of ICU admission and death were 1.5/100,000 and 0.6/100,000 for the total patients, respectively, with the highest value being found in elderly patients aged ≥ 65 years (2.2 and 1.8 per 100,000, respectively).

**Table 1:** Incidence of Hospitalization, ICU Admission and Fatal Outcomes in the 141 Patients With 2009 Pandemic A(H1N1) Laboratory Confirmed Infection

Age group (years)	Population (n)	Hospital admitted n (%)	Incidence per 100,000 inhabitants	ICU admitted	Incidence per 100,000 inhabitants	Death	Incidence per 100,000 inhabitants
0 - 17	136,563	47 (33)	34.4	1	0.7	0	0
18 - 64	589,592	63 (45)	10.7	8	1.4	2	0.3
≥ 65	225,514	31 (22)	13.7	5	2.2	4	1.8
Total	951,669	141 (100)	14.8	14	1.5	6	0.6

### Demographic characteristics and comorbidities

As reported in Table 2, approximately one half of the 141 hospitalized patients were female. The mean age of the overall hospitalized patients A(H1N1) positive examined was 37 years (range 1 month - 93 years) and it was higher in patients admitted to ICU (mean age 35 years for GMW vs 54 years for ICU admitted patients). Of the 141 patients, 78 (55%) had at least an associated medical condition. As compared to those admitted to GMW, patients admitted to ICU presented a higher incidence of cardiac, renal and neurological diseases and of immunocompromission in a statistically significant way. Pregnancy and obesity data, when available, were also examined, because they have been hypothesized to be possible risk factors for the A(H1N1) pandemic virus [5]. No increased risk of poor outcome was observed in the eight pregnant women of the 25 who were of childbearing age (15 - 44 years) and who were hospitalized. Seven of them were admitted to GMW and only one to ICU. Ten patients were reported to be obese, 3 admitted to ICU but obesity was not included in Table 2, since no systematic data were collected on patient height and weight.

**Table 2:** Demographic Characteristics and Comorbidities of Hospitalized Patients With A(H1N1) Laboratory Confirmed Infection and Comparison of Patients in GMW and in ICU

	Hospitalized (n = 141)	GMW* (n = 127)	ICU** (n = 14)	ICU vs GMW P value
Age, mean (range)	37 (1 m - 93 y)	35 (1 m - 93 y)	54 (1 m - 83 y)	0.022
Female	66 (47%)	60 (47%)	6 (43%)	0.755
Comorbidities n (%)^a^				
Any comorbidity	78 (55)	66 (52)	12 (86)	0.016
Respiratory diseases	18 (13)	15 (12)	3 (21)	0.306
Asthma	5 (4)	5 (4)	0 (0)	0.450
Diabetes	21 (15)	17 (13)	4 (29)	0.130
Cardiovascular diseases	33 (23)	26 (20)	7( 50)	0.013
Renal diseases	2 (1)	1 (1)	1 (7)	0.056
Neurological diseases	16 (11)	11 (9)	5 (36)	0.002
Immuno compromised^b^	12 (9)	8 (6)	4 (29)	0.005
Pregnancy^c^	8/25 (32)	7/22 (32)	1/3 (33)	0.5439

GMW: General Medical Ward; ICU: Intensive Care Unit; a: More than one possible medical condition per patient; b: From either medication or immune disorders; c: Calculated as percentages of pregnancy considering the 25 fertile women (15 - 44 years).

### Clinical presentation

Symptoms, laboratory and radiological findings are reported in Table 3. The typical influenza like illness with fever and cough, occasionally accompanied by other classical symptoms (sore throat, myalgia, etc.) was present in most patients and some of them presented gastrointestinal symptoms. Respiratory distress and chest radiography confirmed pneumonia (only 110 patients were examined by X-ray) was observed in 33% and 49% of the people, respectively. Respiratory distress, diarrhea and chest X-ray confirmed pneumonia were found to be more frequent in more severe cases. On the contrary, a sore throat was observed more frequently in people admitted to GMW. The most frequently observed abnormal laboratory findings were anemia, leukocytosis, leucopenia and transaminase alterations. Thrombocytopenia, transaminase and creatine phosphokinase (Cpk) alterations were more frequent in ICU admitted patients as compared with those admitted to GMW.

**Table 3:** Symptoms and Laboratory Findings at Hospital Admission, Clinical Course and Outcome in Hospitalized Patients With A(H1N1) Laboratory Confirmed Infection and Comparison of Patients in GMW and in ICU

	Hospitalized (n = 141)	GMW (n = 127)	ICU (n = 14)	ICU vs GMW P value
Symptoms n (%)				
Fever	137 (97)	124 (98)	13 (93)	0.307
Myalgia	40 (28)	38 (30)	2 (14)	0.218
Cough	110 (78)	100 (79)	10 (71)	0.167
Headache	37 (26)	35 (28)	2 (14)	0.284
Nasal congestion	29 (21)	28 (22)	1 (7)	0.190
Rhinorrhea	19 (13)	17 (13)	2 (14)	0.925
Sore throat	52 (37)	51 (40)	1 (7)	0.015
Respiratory distress	46 (33)	37 (29)	9 (64)	0.008
Diarrhea	19 (13)	14 (11)	5 (36)	0.010
Abdominal pain or vomiting	26 (18)	24 (19)	2 (14)	0.673
Radiological and Laboratory findings n (%)				
Pneumonia seen on chest radiography	54/110 (49)	42/96 (44)	12/14 (86)	0.003
Anemia	81 (57)	70 (55)	11 (79)	0.092
Leukocytosis	50 (35)	42 (33)	8 (57)	0.074
Leukopenia	33 (23)	28 (22)	5 (36)	0.252
Thrombocytosis	19 (13)	16 (13)	3 (21)	0.358
Thrombocytopenia	13 (9)	9 (7)	4 (29)	0.008
Transaminase alteration	39 (28)	31 (24)	8 (57)	0.009
Cpk* alteration	21 (15)	16 (13)	5 (36)	0.021
Pharmacological treatment n (%)				
Antiviral	66 (47)	52 (41)	14 (100)	0.000
Antibiotic	116 (82)	102 (80)	14 (100)	0.067
Corticosteroid	23 (16)	20 (16)	3 (21)	0.585
Course mean days (range)				
Interval from onset of illness to hospital admission	3 (0-14)	3 (0-14)	3 (0-9)	0.855
Hospital stay	9 (1-69)	8 (1-69)	19 (3-59)	0.000
Outcome n (%)				
Survived	135 (96)	127 (100)	8 (57)	0.000
Expired	6 (4)	0	6 (43)	0.000

### Pharmacological therapy

About one half (47%) of the hospitalized case patients received treatment with neuraminidase inhibitors (Table 3). All the people admitted to ICU were treated with antivirals, whereas only 41% of those admitted to GMW received treatment and the differences were statistically significant. However similar values were observed in ICU and GMW admitted patients for the median time from the onset of illness to the initiation of antiviral therapy (5 days, range 0 - 15) and for the patients receiving antiviral therapy within 48 hours after the onset of symptoms (30%) or treatment with antibiotics (82%) and corticosteroids (16%).

### Clinical course and outcome

The median time interval from illness onset to hospital admission was 3 days for all the groups examined. The mean time of hospital stay was 9 days and was statistically shorter for those admitted to GMW as compared to those in the ICU (8 vs 19 days). All patients admitted to GMW were discharged whereas 6 of the 14 patients admitted to ICU had a fatal outcome. The mean age of the 6 patients who died was 70 years (range 49 - 83) and the median time from the onset of illness to death was 15 days (range 5 - 38). All the deceased patients had an underlying medical condition, including, most commonly, cardiovascular diseases (67%), diabetes (50%), chronic obstructive pulmonary disease and neurological disorders (33%) and were treated with antivirals and antibiotics. Pneumonia was detected in 5, respiratory failure in 4, acute respiratory distress syndrome, coma, and important pleural effusion in 2, hypovolemic shock in 1 of the 6 patients.

### Risk factors for admission to ICU

By multivariable logistic regression analysis, an immunosuppression condition was associated to admission to ICU in a statistically significant way, independently of age. The relationship with a poor outcome of the cardiovascular and neurological diseases was confounded by age, which remained per se significantly associated to admission to ICU. Having a positive chest X-ray was associated to the admission to ICU even after adjusting for respiratory symptoms. In univariable analyses, pneumonia and other non-cardiac complications (such as sepsis, ARDS, seizures, coma) were associated with death; when tested together in a bivariable logistic analysis, only “other complications” remained related to the fatal outcome in a statistically significant way.

### Analyses according to age classes

Considering the 141 patients subdivided by age, 47 (33%) were children or adolescents (0 - 17 yrs), 63 (45%) adults (18 - 64 yrs) and 31 (22%) elderly (≥ 65 yrs) (Table 1). The frequency of comorbity was higher in the group of elderly people (≥ 65 yrs, 90%) as compared with the two groups of younger patients (60% for 18 - 64 yrs vs 26% for 0 - 17 yrs). In particular respiratory diseases, diabetes, cardiovascular and neurological diseases were more frequent in the elderly. Comparing symptoms and laboratory findings, the youngest people showed more sore throat and nasal congestion and the elderly more respiratory distress and X ray confirmed pneumonia as compared with people of the other age groups. The incidence of anemia was higher in children, whereas leukocytosis and Cpk levels alterations in the elderly. Non-severe outcome was more frequent in patients aged 0 - 17 (36%) and 18 - 64 (43%) as compared with the oldest people (21%). On the contrary, the percentage of non-fatal ICU admission were higher in the group aged 18 - 64 (75%) as compared with patients aged 0 - 17 (12.5 %) and ≥ 65 (12.5%). Of the 6 people who died after ICU admission, 67% were aged ≥ 65 and 33% 18 - 64 years, whereas all the youngest patients, aged 0 - 17, resolved their infection.

## Discussion

Because of the possibility of the unforeseen emergency of new pandemic influenza viruses it is important to know if it might be possible evaluate the epidemiological and clinical characteristics of a pandemic in a country in the absence of a pre-organized programme. This study represents the first report examining hospitalized people of all age groups with laboratory confirmed A(H1N1) infection during the first A(H1N1) influenza virus pandemic period in Italy and suggests that also a retrospective analysis of a restricted number of people living in a circumscribed area of a country can furnish useful information for evaluating the general characteristics of the 2009 pandemic.

The study deals with patients hospitalized in Umbria, a region of central Italy with a population of less than 1 million people, whereas the total population in Italy exceeds 60 millions, in the period July 2009 - March 2010. The results reported were obtained estimating the clinic-epidemiological features collected examining retrospectively the medical records of 141 patients hospitalized in Umbria with laboratory confirmed 2009 pandemic influenza A(H1N1) infection and are in accordance with those found in other European and non European countries [5].

As observed, in Italy [6] and in other European countries [11-14], the epidemic started almost two months before the usual influenza season. The dates of onset of the cases studied ranged from July 21st to December 16th, 2009, and a peak was reached in November, whereas the seasonal influenza generally peaks in January/February. After the mid-December 2009 there was an abrupt cessation of influenza virus circulation, both inside and outside the hospitals as documented by the local virological surveillance (data not shown).

The pandemic virus was found to be able of inducing severe illness requiring hospitalization in people of different age, even in those with no underlying medical conditions. Although the outbreak affected all the age groups examined, the median age of the hospitalized patients was 37 years, lower than that of patients with seasonal influenza [15] and in accordance with other studies reporting high rates of hospitalization in young adults and in children [5, 11-14], the highest values of hospitalization were observed in the age group 0 - 17 (incidence for 100,000 inhabitants, 34.4 vs 10.7 for age group 18 - 64 and 13.7 for those aged ≥ 65 yrs).

Considering the more severe cases, i.e., people requiring ICU admission, including people who did not survive, the mean age of ICU admitted patients was statistically higher as compared with those admitted to GMW (54 vs 35 yrs) (Table 2). Moreover, in accordance with other data [5, 16-18], the highest absolute number of ICU admission (non-fatal and fatal) was found in people aged 18 - 64 yrs and the lowest in people aged 0 - 17 yrs, whereas the highest number of deaths was observed in the oldest group (≥ 65 yrs). Considering the incidence per 100,000 inhabitants of ICU admission and deaths, the respective values were again lowest in children aged < 18 (0.7 and 0.0), intermediate in the adult aged 18 - 64 (1.4 and 0.3) and highest in those aged 65 yrs and over (2.2 and 1.8) (Table 1).

These data seem to be in contrast with the finding of a higher incidence of hospitalization in the youngest people as compared with the oldest ones. This is probably due to the presence in the elderly of pre-existing antibodies from natural exposure to the A(H1N1) strains that circulated in the years following 1918 or to vaccine induced cross-reactivity [19-21]. A possible explanation of the higher incidence of serious complication in the elderly, ICU admission with death, is suggested in a recent study by Zhou and McElhaney [22]. If pre-existing antibodies are not present or not able to induce a protective immunity in older adults and the new virus can infect them, there is an increased risk of complicated influenza due to the age-related marked decline in memory and effector CTL response to influenza infection.

Similar to data from other studies [5], chronic medical conditions considered to be a risk for seasonal infection were present in some, but not all, hospitalized patients (55%) and the likelihood increased with the age (26% of the patients aged 0 - 17 yrs, 60% in the 18 - 64 yrs group and 90% in the ≥ 65 yrs group) and with severity (86% in people requiring ICU admission and people who did not survive vs 52% in people admitted to GMW). Multivariable analysis confirmed that the presence of co-morbidity, strictly related to age and probably not independently of it, might represent a greater risk factor for ICU admission and death [5, 12, 13].

However, our data were not sufficient to support an association between other risk factors, like pregnancy and obesity, hypothesized to be associated with severe A(H1N1) infections [5]. Since 37.5% of the pregnant women and 90% of the obese patients had co-morbidity factors, it remained unclear whether pregnancy and obesity might be considered risk factors for hospitalization per se. Most patients presented with typical influenza like illness symptoms, although in accordance with previous reports [4], gastrointestinal symptoms were reported in 23% of the patients and in all the age groups examined and severity was found to be associated with respiratory distress and X-ray confirmed pneumonia.

Our data are subjected to limitations. Firstly, we examined a limited number of hospitalized patients, since our study was restricted to Umbria, which geographically limited itself. However, this is the first report examining hospitalized people of all age groups with a laboratory confirmed A(H1N1) infection in Italy, since previously published data are relative to selected groups of population [7-9]. Although the activity of the Italian network for influenza surveillance (INFLUNET) was implemented in the context of pandemics, some cases may have escaped reporting. The data were concerned only hospitalized patients with a laboratory confirmed diagnosis and with an accessible medical chart. All diagnostic testing was clinically driven. Despite the use of a standardized data collection form, not all information was collected for all patients. Finally, the value of our observations is limited by our inability to compare the present data with those of epidemic years, since the hospital-based virological surveillance was introduced for the first time in Italy in 2009.

In conclusion, despite these limitations and although this study was based on a retrospective design and limited to a restricted number of patients, our data confirm those obtained in other published studies on hospitalization and ICU admission following the 2009 pandemic A(H1N1) virus infection [4, 5, 11-18]. The highest incidence of hospitalization was observed in the youngest people, the greatest risk of ICU admission in adults and the highest risk of death in the oldest patients. The role of underlying conditions as a possible risk factor for severe disease was confirmed.
